# Efficacy of Subconjunctivally Applied Everolimus- and Sirolimus-Pretreated MSCs in Preventing Diabetic Retinopathy

**DOI:** 10.1167/tvst.14.9.19

**Published:** 2025-09-15

**Authors:** Ji Seung Jung, Hyo Youn Jo, Jiyi Hwang, Donghee Kim, Myeongjee Kwon, Jungyeon Yong, Haerin Yoon, Hyun Jik Lee, Kyung-Mee Park

**Affiliations:** 1 Laboratory of Veterinary Surgery and Ophthalmology, College of Veterinary Medicine, Chungbuk National University, Cheongju, Republic of Korea; 2 Laboratory of Veterinary Physiology, College of Veterinary Medicine, Chungbuk National University, Cheongju, Republic of Korea

**Keywords:** diabetic retinopathy, mTOR inhibitor, everolimus, sirolimus, mesenchymal stem cells, subconjunctival injection

## Abstract

**Purpose:**

Diabetic retinopathy (DR) is a leading cause of vision impairment in diabetic patients. This study investigates the preventive effects of subconjunctivally applied mesenchymal stem cells (MSCs) pretreated with mammalian target of rapamycin (mTOR) inhibitors on DR.

**Methods:**

Thirty-six male Sprague-Dawley rats were used, with diabetes induced by a single intraperitoneal injection of streptozotocin (STZ) at 55 mg/kg. Four groups were established: diabetic control, MSC-treated, everolimus-pretreated MSC-treated (MSC-E), and sirolimus-pretreated MSC-treated (MSC-S). Each treated group received subconjunctival injections of 1 × 10^6^ MSCs in 30 µL phosphate-buffered saline at 3, 5, and 7 weeks post-STZ. Electroretinography (ERG) was performed at 2 and 8 weeks post-STZ. At 11 weeks, cataract formation, uveitis grading, and retinal histology were assessed.

**Results:**

mTOR inhibitor-pretreated MSCs significantly prevented the decline in retinal function compared to untreated MSCs and diabetic controls. Everolimus-pretreated MSCs tended to show higher ERG flicker amplitudes than sirolimus-pretreated MSCs, although the difference was not statistically significant. Histologically, both pretreated groups showed preserved thickness in the outer plexiform and nuclear layers, suggesting protection against retinal damage.

**Conclusions:**

Subconjunctival application of MSCs pretreated with mTOR inhibitors may help prevent DR progression, with everolimus showing a potential advantage in preserving retinal function.

**Translational Relevance:**

These findings suggest that mTOR inhibitor-pretreated MSCs could be a viable therapeutic strategy for DR, warranting further clinical trials to confirm efficacy and safety in humans.

## Introduction

Diabetic retinopathy (DR) is the most common microvascular complication of diabetes and one of the leading causes of blindness and visual impairment.^1^ DR is classified into nonproliferative diabetic retinopathy (NPDR) and proliferative diabetic retinopathy (PDR), based on the presence or absence of abnormal new blood vessels in the retina.^2^^,^^3^ Chronic hyperglycemia damages the microvasculature of the retina, increasing vascular permeability, which leads to vascular leakage and retinal edema. Simultaneously, there is a loss of retinal endothelial cells and pericytes, accelerating the breakdown of the retinal vascular structure.^4^ Chronic inflammation and oxidative stress play crucial roles in the development and progression of DR. This induces leukostasis, where leukocytes accumulate within the blood vessels, leading to vascular occlusion and hypoxia in the retina. In response to hypoxia, vascular endothelial growth factor (VEGF) is excessively secreted, prompting the formation of abnormal new blood vessels.^5^ In the early stages of DR, pathological changes, such as thickening of the retinal capillary basement membrane, loss of endothelial cells and pericytes, and the formation of microaneurysms, are observed. These changes impair the microcirculation of the retina, reduce oxygen supply to retinal cells, and lead to a decline in retinal function.^3^^,^^6^

There are various treatment options currently available for DR, and the choice of treatment depends on the stage of the disease and the patient's condition. Laser photocoagulation uses laser energy to destroy abnormal blood vessels and prevent bleeding. Anti-VEGF therapy inhibits the formation of new blood vessels, and steroid injections reduce retinal edema.^3^^,^^7^ These injections are typically administered through intravitreal injection; however, repeated intraocular injections can lead to various complications, including cataracts, retinal detachment, retinal hemorrhage, and endophthalmitis.^8^^,^^9^ Additionally, frequent injections may impose an economic burden. Previous studies using subconjunctival injections have demonstrated therapeutic effects on the posterior segment,^10^^,^^11^ and our own research has confirmed the effectiveness of subconjunctival injections in reaching the retina.^12^^–^^14^

Recent research on treatments for DR has focused on using mesenchymal stem cells (MSCs).^15^^–^^19^ MSCs can be derived from various tissues and possess anti-inflammatory and immunomodulatory properties, making them promising candidates for DR treatment.^20^^,^^21^ MSCs help regenerate damaged retinal tissue, reduce inflammation, and regulate the expression of angiogenic factors like VEGF, thereby inhibiting the formation of abnormal new blood vessels.^22^ Additionally, MSCs interact with surrounding cells, such as pericytes, through various biological signals to promote tissue repair. Currently, MSC-based therapies are primarily in the preclinical research stage using animal models, with some early-phase clinical trials underway.^21^^,^^23^^,^^24^

However, the efficacy of MSCs is diminished under hyperglycemic conditions due to several complex factors. High glucose levels lead to the excessive generation of reactive oxygen species (ROS), increasing intracellular oxidative stress, which impairs MSC survival and function, particularly reducing their proliferation and differentiation capacity. Additionally, the expression of inflammatory cytokines such as TNF-α and IL-1β is elevated, further inhibiting MSC function and reducing cell survival rates. Moreover, the accumulation of advanced glycation end-products increases cell apoptosis, collectively diminishing the therapeutic efficacy of MSCs in hyperglycemic environments.^25^ Therefore, strategies to reduce oxidative stress and suppress inflammatory responses are necessary to effectively utilize MSCs under hyperglycemic conditions. The key to enhancing cell survival is to activate cell autophagy. Autophagy is a self-digestion process where cells remove and recycle unnecessary or damaged components to maintain cellular homeostasis.^26^ It plays a crucial role in maintaining cellular metabolism and function, regulating growth and differentiation, and preventing ROS-induced senescence.^27^^,^^28^ Sirolimus and everolimus are drugs used to suppress immune responses following organ transplantation or in the fabrication of drug-eluting stents. They function by inhibiting the mammalian target of rapamycin (mTOR). Everolimus has an additional 2-hydroxyethyl group at the C40 position, which distinguishes it from sirolimus and results in distinct pharmacokinetic and pharmacodynamic properties.^29^ Sirolimus and everolimus inhibit mTOR signaling in MSCs, enhancing their immunosuppressive properties and promoting autophagy, which improves the survival rate of transplanted MSCs. Additionally, some studies suggest that mTOR inhibition enhances cell differentiation potential and reduces cellular senescence.^30^^–^^32^ While the transplantation of MSCs pretreated with sirolimus, based on the fact that mTOR inhibitors are potent activators of autophagy, has been evaluated,^31^^,^^33^ studies on the efficacy of MSCs pretreated with everolimus and comparative studies with sirolimus are lacking.

In this study, we investigated whether untreated MSCs, as well as MSCs pretreated with sirolimus and everolimus, can prevent the progression of diabetic retinopathy when applied subconjunctivally in diabetic rats.

## Materials and Methods

### Cell Culture

Human umbilical cord blood–derived mesenchymal stem cells (hUCB-MSCs) were obtained from CEFO Co. (Seoul, South Korea). Fetal bovine serum (FBS) was purchased from Thermo Fisher Scientific (Waltham, MA, USA). Minimal essential medium alpha modification (α-MEM) was obtained from Hyclone (Logan, UT, USA). D-glucose was purchased from Sigma-Aldrich (St. Louis, MO, USA). hUCB-MSCs were cultured in KSB-3 Complete Medium Kit (Kang Stem Biotech, Seoul, South Korea) with 10% FBS in a 5% CO₂ environment at 37°C.

### Water-Soluble Tetrazolium Salt Cell Proliferation Assay

EZ-Cytox enhanced cell viability assay kit (DoGenBio, Seoul, Korea) was used for cell viability assessment. hUCB-MSCs were seeded in 96-well plates at a concentration of 1.0 × 10^4^/well. To distinguish between the control and high glucose groups, 0 and 25 mM of D-glucose were used, respectively. After exposure to high glucose and everolimus and sirolimus for 72 hours, the cell supernatant was removed. Then, 90 µL α-MEM medium per well was mixed with 10 µL EZ-Cytox reagent and incubated for 3 hours at 37°C in 5% CO_2_. The absorbance was measured at 450 nm.

### RNA Isolation and Reverse Transcription Polymerase Chain Reaction

A solution of 50× dithiothreitol diluted with RL buffer was used to lyse the cells. An RNA extraction kit (TaKaRa, Shiga, Japan) was used to extract messenger RNA (mRNA) from the samples. Then, 1 µg mRNA was put into a reverse transcription polymerase chain reaction (PCR) premix tube (iNtRON biotechnology, Seongnam, Korea) and reverse transcribed into complementary DNA (cDNA). The process was performed for 1 hour at 45°C, followed by 5 minutes at 95°C.

### Real-Time Quantitative Polymerase Chain Reaction

The cDNA was amplified with a TB green Premix Ex Taq (TaKaRa, Otsu, Japan). The mRNA expression levels of the *NT5E* (encoding CD73), *THY1* (encoding CD90), *ENG* (encoding CD105), and *ACTB* genes were determined using the CFX connect real-time PCR direction system (Bio-Rad, Hercules, CA, USA) with cDNA samples and primer mixture. For normalization of each gene, we used the gene expression levels of *ACTB*. Quantitative PCR was performed for 10 minutes at 95°C for DNA polymerase activation, with the cycle repeated for 15 seconds at 94°C, 20 seconds at 55°C, and 30 seconds at 72°C for a total of 50 cycles. The primer sequences are listed in Supplementary Table S1.

### Animals

Thirty-six male Sprague-Dawley rats, aged 8 weeks, were obtained from Nara Biotech (Pyeongtaek, Republic of Korea) for this study. The animals were housed under conventional conditions with a standard 12-hour light/12-hour dark cycle. They had free access to normal pellet chow (Experimental Rat & Mouse Diet; Purina, St. Louis, MO, USA) and water. The experimental protocol was approved by the Institutional Animal Care and Use Committee at Chungbuk National University (IACUC number: CBNUA-2032-22-01) and conducted in accordance with the ARVO Statement for the Use of Animals in Ophthalmic and Vision Research.

### Experimental Design and Grouping

**Figure 1.**
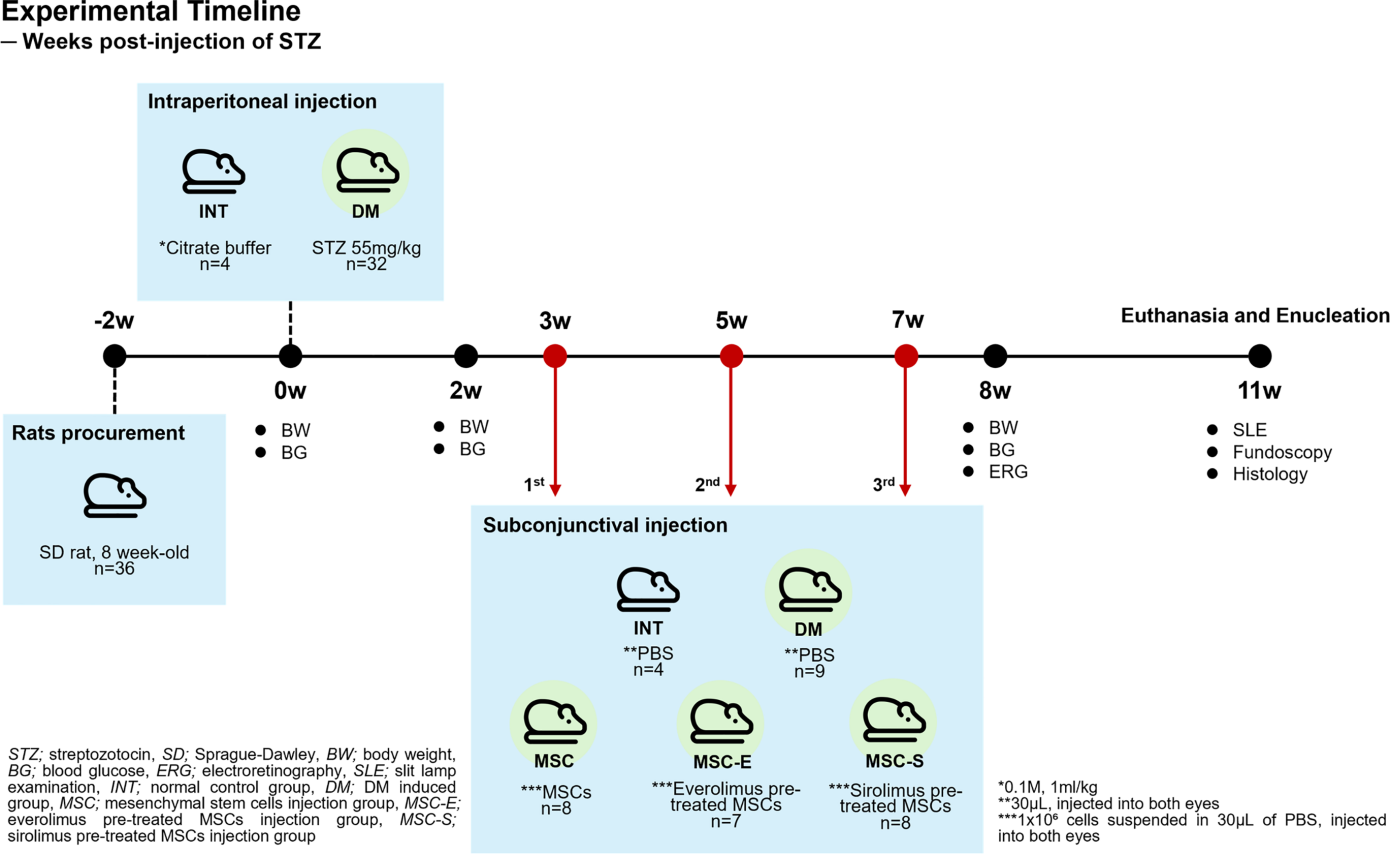
Experimental timeline. The sequence of treatments and measurements is represented according to the weeks following STZ injection.

To induce diabetes, a single dose of 55 mg/kg streptozotocin (STZ; Sigma-Aldrich) in citrate buffer (pH 4.5; Sigma-Aldrich) was administered via intraperitoneal injection. The intact control group (INT, four rats, eight eyes) received an equivalent volume of citrate buffer via intraperitoneal injection. Diabetes mellitus (DM) was confirmed 14 days post-STZ injection when blood glucose levels exceeded 250 mg/dL, as measured by a commercial blood glucose meter (FORA G11; ForaCare, Moorpark, CA, USA). Thirty-two diabetic rats were then randomly divided into four groups: the DM group (DM, 9 rats, 18 eyes), the group receiving subconjunctival MSC injections (MSC, 8 rats, 16 eyes), the group receiving subconjunctival everolimus-pretreated MSC injections (MSC-E, 7 rats, 14 eyes), and the group receiving subconjunctival sirolimus-pretreated MSC injections (MSC-S, 8 rats, 16 eyes). The overall experimental timeline is shown in Figure 1.

### Body Weight and Blood Glucose Measurement

Body weight (BW) and blood glucose (BG) levels were measured after a 6-hour fasting period. These measurements were taken on the day of STZ injection and at 2 weeks and 8 weeks after diabetes induction. Rats that lost more than 30% of their initial body weight were euthanized, and their data were excluded.

### Preparation and Injection of MSCs

For the pretreated MSC groups, MSCs were incubated with 100 nM of either everolimus (Sigma-Aldrich) or sirolimus (Sigma-Aldrich). These solutions were prepared by first diluting the drugs to 10 mM in DMSO and then further diluting them to 100 nM in phosphate-buffered saline (PBS). MSCs were pretreated with these solutions for 24 hours. Subconjunctival injections were performed using an insulin syringe, with 1 × 10⁶ cells suspended in 30 µL PBS injected into both eyes of each rat. The MSC group received MSCs. The MSC-E and MSC-S groups received everolimus-pretreated MSCs and sirolimus-pretreated MSCs, respectively. The INT group and the DM group received an equivalent volume of PBS. The procedure was conducted under isoflurane inhalation anesthesia and proparacaine topical anesthesia, with the injection sites disinfected using 0.5% povidone–iodine solution. Injections were administered three times: at 3 weeks, 5 weeks, and 7 weeks post-STZ injection.

### Electroretinography

Electroretinography (ERG) recording was conducted at 8 weeks post-STZ injection using the RETevet ERG system (LKC, Gaithersburg, MD, USA). Pupils were dilated using 0.01% tropicamide and phenylephrine (Mydrin-P; Santen, Osaka, Japan), and topical anesthesia was applied before the examination. All rats underwent ERG recording in dim light conditions after a dark adaptation period of 20 to 30 minutes. The flash conditions were set to 8.0 cd × s/m^2^ at 2 Hz, and flicker conditions were set to 28.3 Hz. All procedures were performed under intermittent isoflurane inhalation anesthesia, with anesthesia depth monitored throughout each procedure.

### Additional Ocular Assessments: Slit-Lamp Examination and Fundoscopy

At 11 weeks post-STZ injection, slit-lamp examination and fundoscopy were performed under intermittent isoflurane anesthesia, as previously described. The slit-lamp exam was used to grade and compare the stages of uveitis and cataracts among the groups. The evaluation criteria, adapted from the SPOTS system,^34^ are provided in Tables 1 and 2. Slit-lamp examination was conducted using the MW50D (Grand Seiko Co. Ltd., Hiroshima, Japan), and fundoscopy was performed with an indirect ophthalmoscope (Vantage Plus, Keeler Ltd., Windsor, UK) with a 28-diopter lens (Volk Optical, Mentor, OH, USA). Afterward, all rats were euthanized, and their eyes were enucleated for histologic analysis.

**Table 1.** Criteria for Cataract Evaluation

Grade		Cataract
0	Normal	No cataract material, red reflex present
1	Incipient	<10% cataract material visible on retro illumination, red reflex present
2	Early immature	10% to 50% cataract material visible on retro illumination, red reflex present
2.5	Late immature	50% to 99% cataract material visible on retro illumination, weak red reflex
3	Mature	100% cataract material visible on retro illumination, no red reflex

**Table 2.** Criteria for Uveitis Evaluation

Grade	Uveitis
0	No congestion in iris blood vessels
1	Mild congestion of secondary vessels, no congestion in tertiary vessels
2	Mild congestion of tertiary vessels, moderate congestion of secondary vessels
3	Moderate congestion of both secondary and tertiary vessels, or mild edema in the iris stroma

### Histologic Analysis

At 11 weeks post-STZ injection, all rats were euthanized via inhalation of high-dose isoflurane until loss of vital signs, followed by thoracotomy, in compliance with American Veterinary Medical Association (AVMA) guidelines. Their eyes were enucleated and fixed in BioFix HD (BioGnost, Zagreb, Croatia). Each eye, including the optic nerve, was bisected sagittally to create two hemispheres. The lens and vitreous humor were removed. After routine tissue processing and paraffin embedding, sections were cut at a thickness of 5 µm and stained with hematoxylin and eosin. Histologic images were acquired using the KB-600 biological microscope (Korea Lab Tech, Hwaseong-si, Korea) and Optiview (Korea Lab Tech., Hwaseong-si, South Korea) imaging software. Retinal thickness measurements were performed using ImageJ (National Institutes of Health, Bethesda, MD, USA).

For each group, both eyes from four rats (*n* = 8 eyes/group) were used for analysis. Measurements were taken from the same distance from the optic nerve head, using sections where the optic nerve was visible, to maintain consistency in retinal region selection.

### Statistical Analysis

Statistical analyses and graphing were conducted using SigmaPlot 12.0 software (Systat Software, San Jose, CA, USA), Prism 10 software (GraphPad Software, La Jolla, CA, USA), and IBM SPSS Statistics version 31 (IBM Corp., Armonk, NY, USA). All quantitative data were expressed as mean ± standard error of the mean. Comparisons between two experimental groups were performed by a two-tailed Student's *t*-test, and comparisons between three or more experimental groups were evaluated using one-way analysis of variance. For data sets involving repeated measures from both eyes of the same animal, a linear mixed model (LMM) was applied with Rat ID as a random effect and group as a fixed effect, followed by Bonferroni-corrected post hoc comparisons. A *P* value of less than 0.05 was considered to indicate statistical significance.

## Results

### Effect of mTOR Inhibitors Under High Glucose Conditions

**Figure 2.**
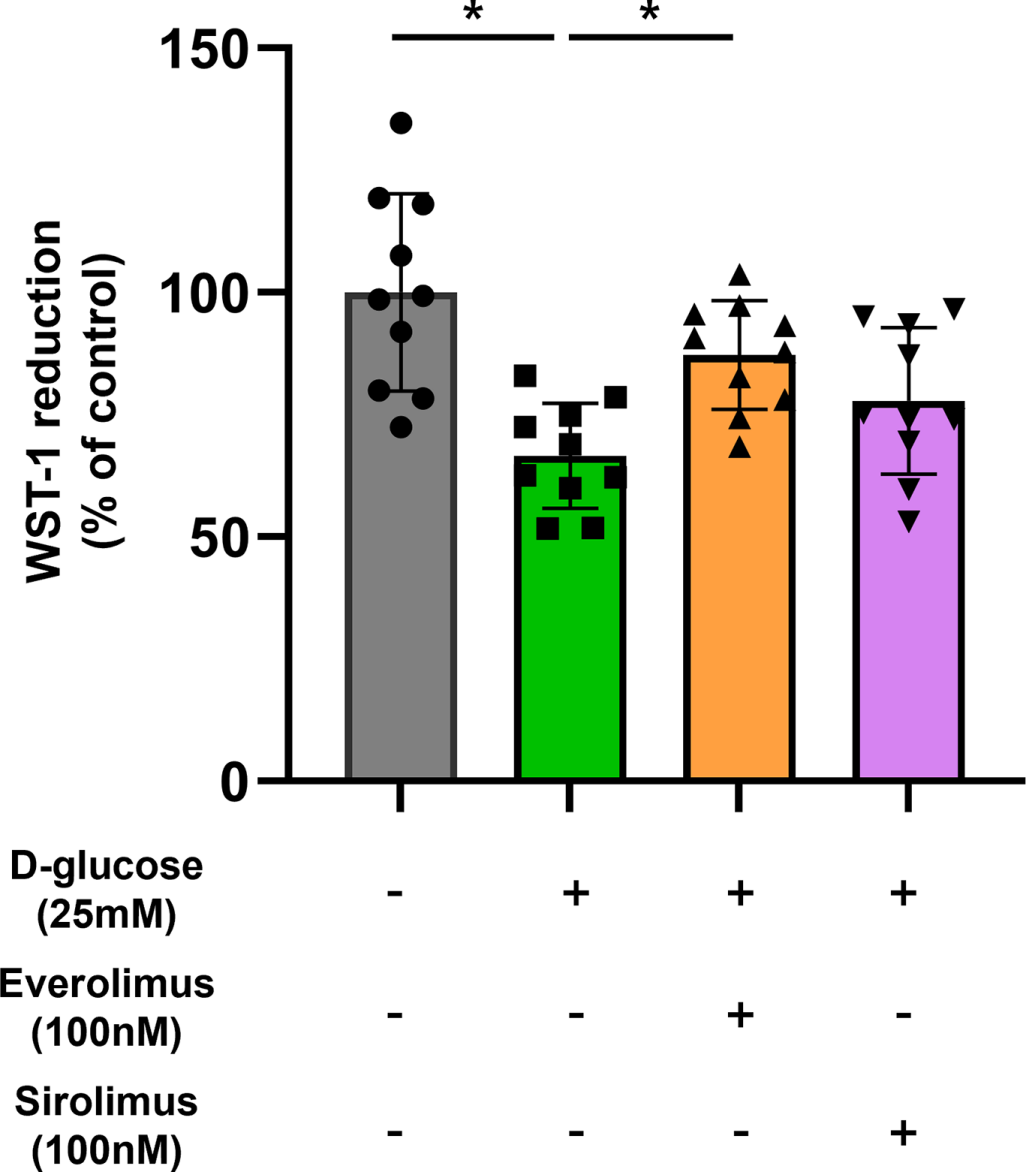
Effect of mTOR inhibitors on MSCs under high glucose conditions. Cells were treated with everolimus (100 nM) or sirolimus (100 nM) in the presence of D-glucose (25 mM) for 72 hours. The WST-1 cell proliferation assay was used to determine cell viability. Quantitative data are displayed as mean ± standard deviation using a scatterplot. *P < 0.05. WST-1, water-soluble tetrazolium salt.

First, we performed the water-soluble tetrazolium salt assay to measure cell viability of MSCs under high glucose conditions. The result showed a decrease in cell proliferation in the high glucose exposure group compared to the control group. Furthermore, high glucose–reduced survival of MSCs was statistically recovered by the everolimus pretreatment. The sirolimus-pretreated group exhibited a marginal increase in mean survival relative to the high glucose group, but this difference was not statistically significant (Fig. 2).

### Effect of mTOR Inhibitors’ Authentication Marker Expression

**Figure 3.**
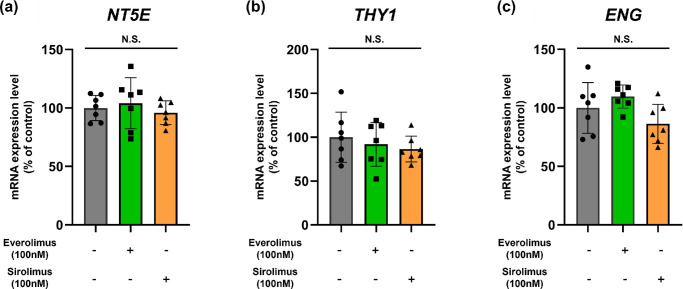
Effects of mTOR inhibitors on MSC authentication marker expression. (**a–c**) Cells were treated to either everolimus (100 nM) or sirolimus (100 nM) for 24 hours. The mRNA levels of *NT5E* (CD73), *THY1* (CD90), and *ENG* (CD105) were analyzed by real-time quantitative PCR. Quantitative data are presented as mean ± standard deviation using a scatterplot. N.S. indicates not statistically significant.

Next, we investigated the effect of the drugs on the function of MSCs: treatment with both everolimus and sirolimus did not affect the mRNA levels of MSC-specific stem cell markers (Fig. 3). It has been well documented that MSCs exhibit elevated expression levels of CD73 (*NT5E*), CD90 (*THY1*), and CD105 (*ENG*).^35^^,^^36^ The stemness of MSCs is preserved after drug treatment regardless of the presence of FBS, at the time of cell transplantation.

### Assessment of BW and BG

**Figure 4.**
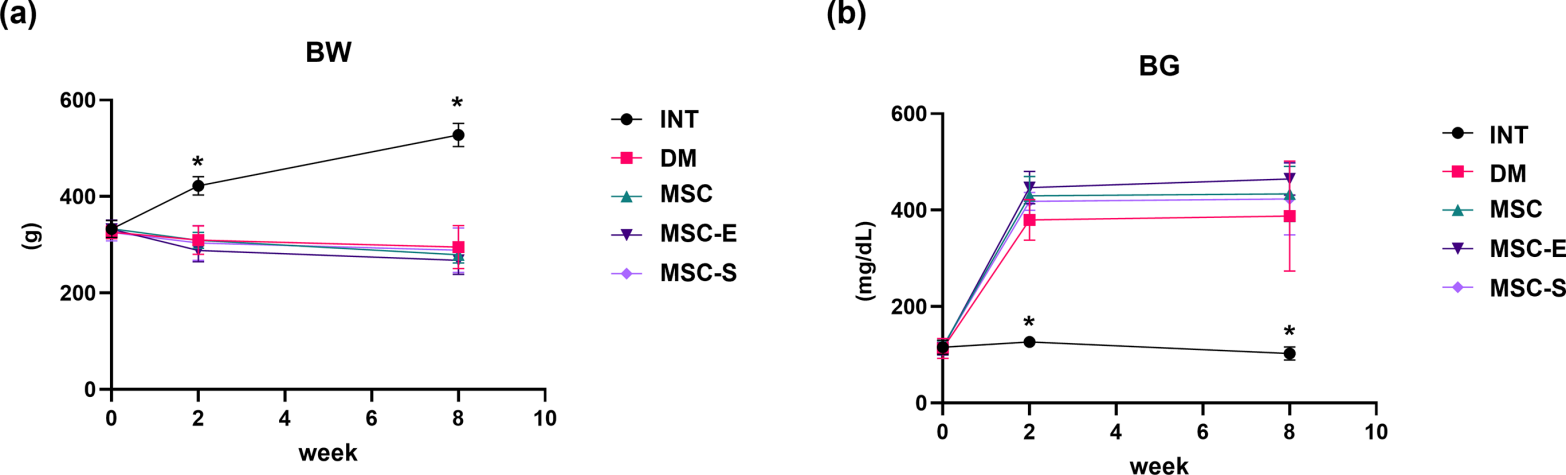
(**a**) Changes in BW over weeks following STZ injection. (**b**) Changes in BG over weeks following STZ injection. *P < 0.05.

At the time of STZ injection, there were no significant differences in BW and BG levels among the groups. Two weeks post-STZ injection, the INT group showed significantly higher BW and lower BG levels compared to all diabetic groups, with all *P* values < 0.001. These trends remained consistent at 8 weeks post-STZ injection, with the INT group maintaining significantly higher BW and lower BG levels than the other groups (Fig. 4). No significant differences were observed among the diabetic groups themselves at either time point. The mean ± standard deviation values for BW and BG levels at each time point are presented in Supplementary Tables S2 and S3.

### ERG Evaluation

**Figure 5.**
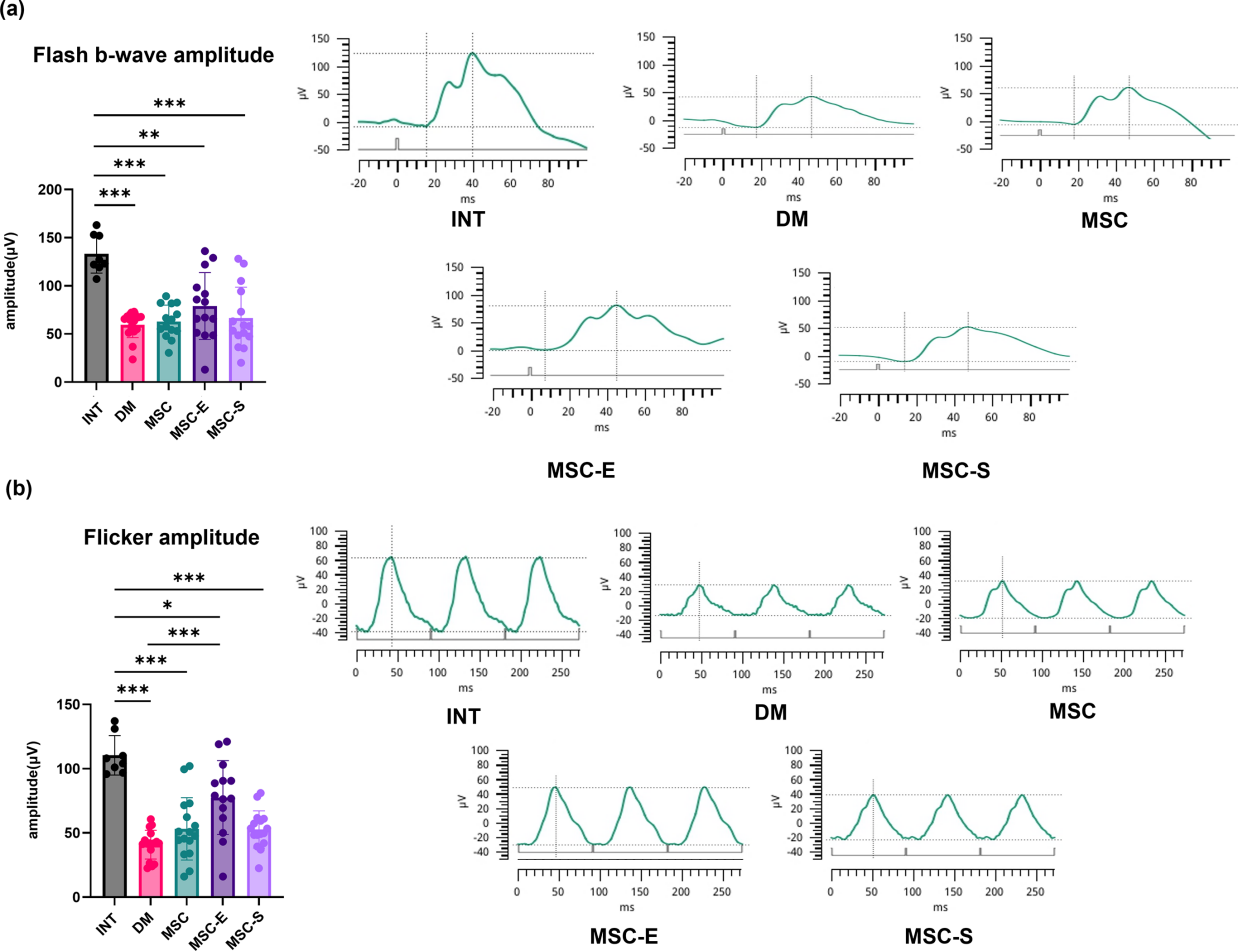
(**a**) Comparison of b-wave amplitude in ERG flash stimulation conducted at week 8 post-STZ injection across different groups. (**b**) Comparison of amplitude in ERG flicker stimulation conducted at week 8 post-STZ injection across different groups. *P < 0.05. **P < 0.01. ***P < 0.001.

In the flash recordings, the estimated mean b-wave amplitudes ± standard error were 133.25 ± 9.83 µV for the INT group, 57.32 ± 6.55 µV for DM, 63.25 ± 7.34 µV for MSC, 78.97 ± 7.43 µV for MSC-E, and 67.03 ± 7.14 µV for MSC-S. LMM analysis revealed that all diabetic groups showed significantly lower b-wave amplitudes compared to the INT group (*P* < 0.001 for DM, MSC, and MSC-S; *P* = 0.001 for MSC-E). However, there were no statistically significant differences among the diabetic groups themselves, although the MSC-E group showed a relatively higher trend compared to the others (Fig. 5a). In the flicker recordings, the estimated mean amplitudes ± standard error were 110.45 ± 8.07 µV for the INT group, 40.87 ± 5.49 µV for DM, 53.18 ± 5.71 µV for MSC, 77.52 ± 6.10 µV for MSC-E, and 52.66 ± 5.71 µV for MSC-S. Flicker amplitudes were significantly reduced in all diabetic groups compared to the INT group (*P* < 0.001 for DM, MSC, and MSC-S; *P* = 0.028 for MSC-E). The MSC-E group exhibited significantly higher amplitudes than the DM group (*P* < 0.001) and showed a trend toward higher amplitudes compared to the MSC and MSC-S groups, although these differences were not statistically significant (*P* = 0.066 and 0.057, respectively) (Fig. 5b). Groupwise mean differences and standard errors for the flash b-wave and flicker amplitudes, based on Bonferroni-corrected comparisons, are summarized in Tables 3 and 4, respectively.

**Table 3.** Bonferroni-Corrected Groupwise Mean Differences and Standard Errors for Flash b-Wave Amplitudes

	INT	DM	MSC	MSC-E	MSC-S
INT	—	75.933 ± 9.908^*^	69.998 ± 12.265^*^	54.279 ± 12.322^*^	66.218 ± 12.147^*^
DM	−75.933 ± 9.908^*^	—	−5.935 ± 9.837	−21.655 ± 9.908	−9.716 ± 9.688
MSC	−69.998 ± 12.265^*^	5.935 ± 9.837	—	−15.720 ± 10.442	−3.781 ± 10.234
MSC-E	−54.279 ± 12.322^*^	21.655 ± 9.908	15.720 ± 10.442	—	11.939 ± 10.302
MSC-S	−66.218 ± 12.147^*^	9.716 ± 9.688	3.781 ± 10.234	−11.939 ± 10.302	—

* *P* < 0.05.

**Table 4.** Bonferroni-Corrected Groupwise Mean Differences and Standard Errors for Flicker Amplitudes

	INT	DM	MSC	MSC-E	MSC-S
INT	—	69.579 ± 9.766^*^	57.269 ± 9.888^*^	32.929 ± 10.121^*^	57.788 ± 9.888^*^
DM	−69.579 ± 9.766^*^	—	−12.311 ± 7.923	−36.651 ± 8.212^*^	−11.792 ± 7.923
MSC	−57.269 ± 9.888^*^	12.311 ± 7.923	—	−24.340 ± 8.357	0.519 ± 8.074
MSC-E	−32.929 ± 10.121^*^	36.651 ± 8.212^*^	24.340 ± 8.357	—	24.859 ± 8.357
MSC-S	−57.788 ± 9.888^*^	11.792 ± 7.923	0.519 ± 8.074	24.859 ± 8.357	—

* *P* < 0.05.

### Histologic Evaluation of the Retina

**Figure 6.**
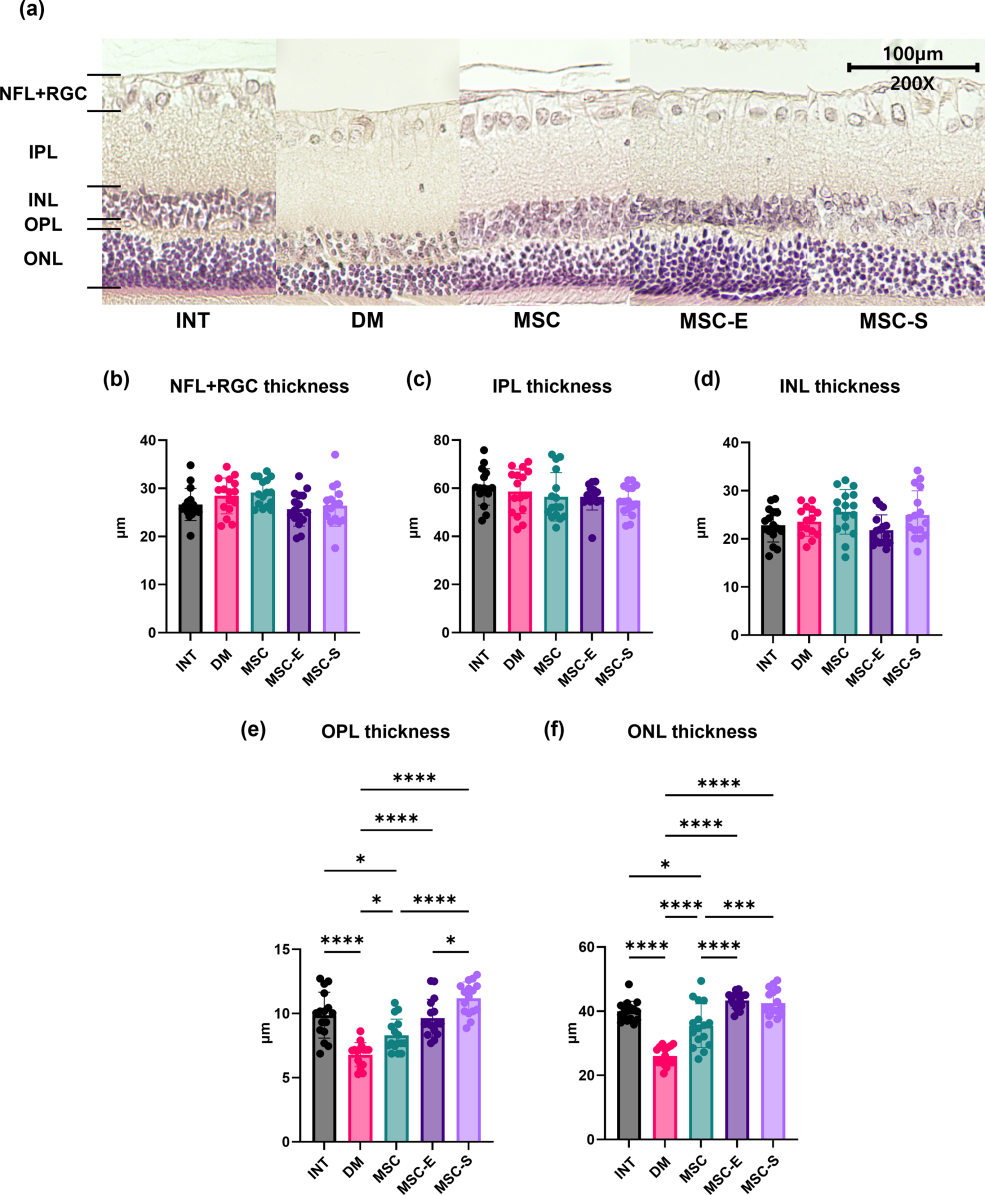
Histologic evaluation of the retina using H&E staining at week 11 post-STZ injection. (**a**) H&E-stained histologic images of the retina from each group at 11 weeks after diabetes induction. (**b–f**) Groupwise comparison of absolute thickness values for each retinal layer: (**b**) NFL + RCG, (**c**) IPL, (**d**) INL, (**e**) OPL, and (**f**) ONL. H&E, hematoxylin and eosin. *P < 0.05. **P < 0.01. ***P < 0.001. ****P < 0.0001.

The thicknesses of individual retinal layers, including nerve fiber layer (NFL) + retinal ganglion cell layer (RGC), inner plexiform layer (IPL), inner nuclear layer (INL), outer plexiform layer (OPL), and outer nuclear layer (ONL), were compared across groups (Fig. 6). There were no significant differences among groups in the NFL + RGC, IPL, and INL. In contrast, the OPL and ONL showed significant groupwise differences. For the OPL, the DM group exhibited significantly reduced thickness compared to the INT, MSC, MSC-E, and MSC-S groups (all *P* < 0.05), with the most marked reductions observed in comparisons with the INT and MSC-S groups (*P* < 0.0001). The MSC-S group was significantly thicker than the MSC and MSC-E groups (*P* < 0.0001 and *P* = 0.0168, respectively), while no significant differences were observed between the INT and MSC-E groups. For the ONL, the INT group was significantly thicker than the DM and MSC groups (*P* < 0.0001 and *P* = 0.0297, respectively), while the MSC-E and MSC-S groups were also significantly thicker than the DM group (both *P* < 0.0001). Additionally, the MSC-E and MSC-S groups were significantly thicker than the MSC group (*P* < 0.0001 and *P* = 0.0001, respectively). No significant difference was found between the MSC-E and MSC-S groups.

### Evaluation of Cataract, Uveitis, and Fundoscopy Findings

**Figure 7.**
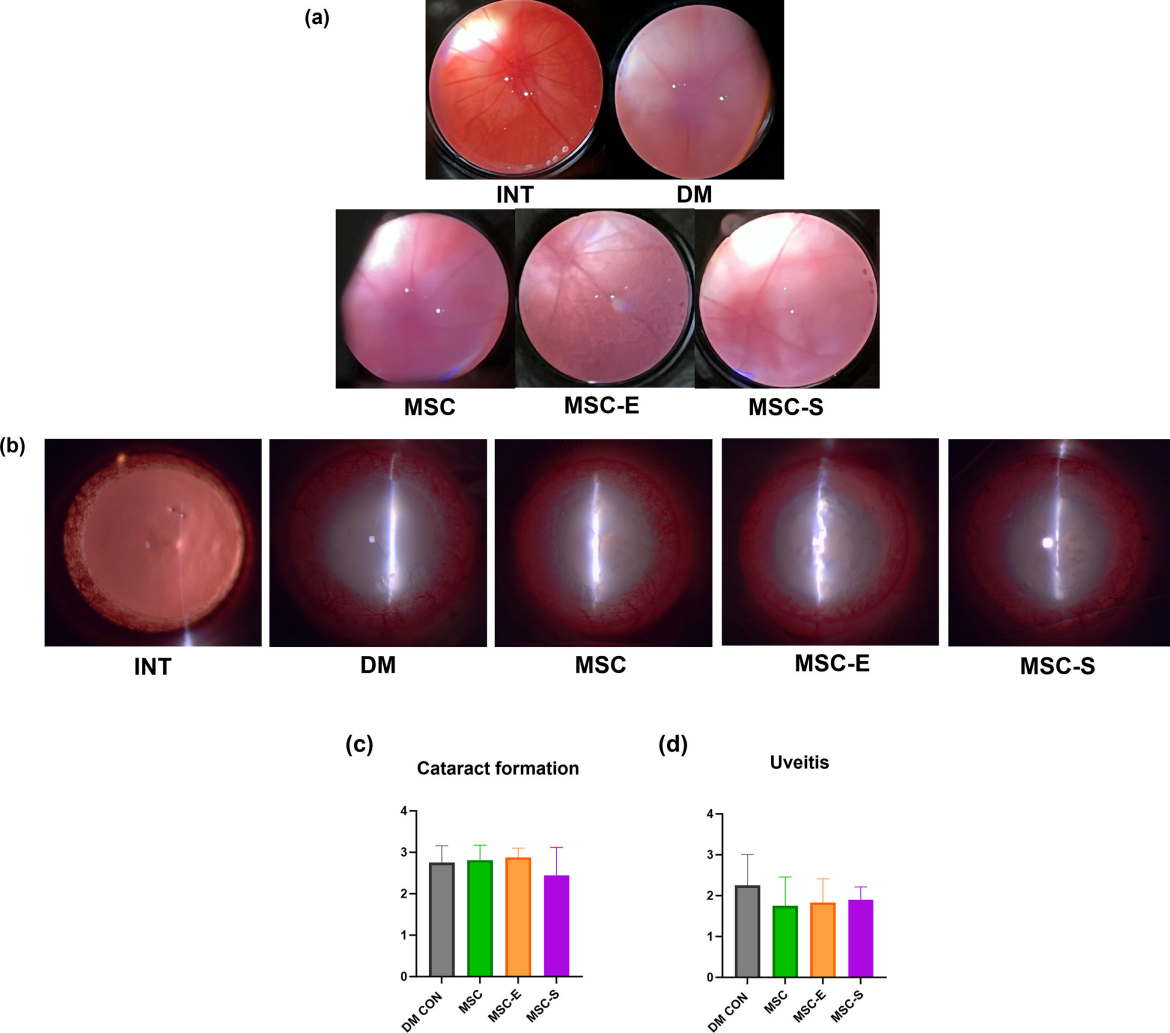
(**a**) Fundoscopic evaluation of retinal vessels and lens clarity. (**b–d**) Evaluation of the lens and iris. Grading is based on the criteria described in Tables 1 and 2.

Cataract formation was observed in all groups except the INT group, with no significant differences between groups, and all groups had cataracts ranging from stages 2 to 3 (Figs. 7b, 7c). Uveitis was also observed in all groups except the INT group, with no significant differences between groups (Figs. 7b, 7d). All rats in the INT group showed no signs of cataract or uveitis upon silt-lamp examination. In the fundoscopy, rats with severe cataracts preventing fundus observation were excluded. The INT group displayed well-formed retinal vessels, while the other groups, particularly the DM group, showed relatively atrophied vessels (Fig. 7a).

## Discussion

This study investigated the preventive effects of subconjunctivally injected MSCs pretreated with mTOR inhibitors everolimus and sirolimus on the progression of DR. Current treatments for DR, such as laser photocoagulation, intravitreal anti-VEGF injections, and steroid injections, primarily manage symptoms and prevent further vision loss.^37^ However, these methods are not fundamental cures but rather focus on alleviating symptoms, making repeated intravitreal injections inevitable. As a result, the economic burden increases, along with the risks of complications such as cataract formation and tractional retinal detachment.^37^^–^^39^ The therapeutic use of MSCs extends beyond retinal diseases, showing promise in treating degenerative, immunogenic, and inflammatory conditions in various organs.^40^^–^^42^ Although the exact mechanisms of MSC therapy are not fully understood, recent evidence suggests that the healing effects are more likely due to paracrine effects, such as cell-derived exosomes, rather than the previously assumed theory of direct differentiation into damaged cells at the target organ.^43^^,^^44^ One study demonstrated that hyaluronic acid–coated nanoparticles administered via subconjunctival injection in mice effectively delivered the drug to the posterior segment of the eye.^11^ Therefore, in this study, we assumed that the factors derived from MSCs would be delivered to the posterior segment; as in our previous studies,^12^^–^^14^ the subconjunctival injection method was employed.

ERG was used to evaluate retinal function, focusing on both flash ERG and flicker ERG responses. The b-wave of flash ERG primarily reflects the combined activity of photoreceptors and downstream bipolar cells, whereas flicker ERG is more specifically associated with cone photoreceptor–bipolar cell pathway function.^45^ Further, in human patients with DM who have mild DR or even no apparent DR, a significant reduction in the flicker ERG amplitude has been observed. This finding suggests that flicker ERG is useful in detecting early dysfunction in diabetes.^46^ In this study, the ERG evaluation showed no significant differences in the flash ERG b-wave amplitudes among the experimental groups. However, in the flicker stimulus, the MSC-E group showed a trend toward a higher amplitude compared to the MSC-S group. This indicates a potential benefit of everolimus pretreatment in preserving cone–bipolar pathway function, although the difference did not reach statistical significance in our analysis (*P* = 0.057). In other words, everolimus-pretreated MSCs tended to prevent flicker amplitude decline more effectively than sirolimus-pretreated MSCs, but this did not meet the threshold for significance. Meanwhile, the ERG a-wave measurements did not reveal significant differences between the groups, and similar findings have been reported in previous studies. These studies have shown that a-wave abnormalities are less pronounced compared to b-wave changes in early DR,^47^^,^^48^ which may explain the lack of significant a-wave differences in our results. Furthermore, since the rat retina is rod-dominant, a distinct negative a-wave deflection is not typically observed, which could also account for these findings.^49^^,^^50^

Histologic analysis of the retina provided structural correlates to the functional findings. We measured absolute thickness of each retinal layer and found that both the OPL and ONL were better preserved in the MSC-E and MSC-S groups compared to DM and MSC groups. This indicates that pretreatment with mTOR inhibitors increased the viability and function of MSCs. Beyond the OPL and ONL, we did not observe a notable difference in the thickness of other retinal layers between any of the treated groups and the diabetic controls. This could be due to the relatively early stage of DR examined in our model, wherein dramatic inner retinal changes might not yet have manifested or might be subtle. While changes in the inner retina are more widely recognized in DM studies,^51^ our study demonstrated significant and distinct differences between the groups, specifically in the outer retina. Whether or not cells in the outer retina, especially photoreceptors, undergo apoptosis in DM remains a subject of conflicting findings across various studies.^52^ A study observed photoreceptor apoptosis starting at week 4 after diabetes induction, with an increase in apoptotic photoreceptors over time. By week 24, only half of the normal cell layer in the ONL remained.^53^^,^^54^ In another study, it was observed that at week 12 after diabetes induction, the outer segments of rods, most middle-wave cones and some short-wave cones, had morphologically degenerated.^53^^,^^55^ Our results are consistent with previous reports that the outer retina is adversely affected by diabetes and, importantly, suggested that MSC therapy with mTOR inhibitor pretreatment may help mitigate this outer retinal degeneration. The preservation of OPL and ONL thickness in the treated groups may contribute to the functional improvements observed in ERG measurements.

In early stem cell research, it was believed that stem cells would home to the target tissue and differentiate to replace damaged cells. However, recent studies indicate limitations in homing/engraftment.^56^ Instead, their beneficial effects are thought to arise from the paracrine mechanism, where they transport helpful substances like cytokines, growth factors, and extracellular vesicles that support tissue repair and modulate inflammation. Thus, strategies to enhance the survival, secretory activity, and overall efficacy of MSCs are crucial for improving therapeutic outcomes. It is known that pretreating MSCs with mTOR inhibitors enhances stem cell survival and efficacy. In our previous experiments using sirolimus-pretreated MSCs in a DR rat model, we observed that MSC treatment led to significant improvements in retinal function and structure compared to untreated diabetic controls.^14^ Specifically, the sirolimus-pretreated MSC group in that study showed flash b-wave amplitudes nearly restored to normal levels and higher flicker amplitudes compared to the non-pretreated MSC group, as well as thicker retinal layers on histology compared to diabetic controls. Those results demonstrated the promise of mTOR inhibition.

In the present study, we extended this approach by comparing sirolimus versus everolimus pretreatment of MSCs, since, to the best of our knowledge, no prior studies have directly investigated everolimus-pretreated MSCs for DR. Everolimus is a derivative of sirolimus designed to have improved pharmacokinetic properties, including faster absorption, higher bioavailability, and a more stable drug concentration in vivo.^29^^,^^57^^,^^58^ These advantages could, in theory, enhance the therapeutic potency of MSCs.

We hypothesized that everolimus-pretreated MSCs might therefore perform better than sirolimus-pretreated MSCs in slowing DR progression. The findings from our study showed that both pretreatment strategies were beneficial: both MSC-E and MSC-S groups exhibited better functional (ERG) outcomes and structural preservation than diabetic rats treated with untreated MSCs. When comparing everolimus versus sirolimus directly, the MSC-E group did show slightly better outcomes, but these differences were not statistically significant. Thus, we cannot conclusively state that everolimus is superior to sirolimus in this setting, based on the current data. Nonetheless, the results suggest a trend favoring everolimus. Importantly, our data confirm that pretreating MSCs with either mTOR inhibitor enhances their therapeutic effect in early DR, as both pretreated groups outperformed the non-pretreated group. Further studies with expanded sample sizes and longer follow-up durations are warranted to more definitively evaluate the comparative efficacy of sirolimus- and everolimus-pretreated MSCs.

This study had the limitation of observing the effects of drug-pretreated MSCs only in the early stages of DR progression due to the systemic condition of the model. Considering that VEGF mRNA expression increases after 3 to 4 months or 30 weeks following diabetes induction,^59^^,^^60^ further studies are needed to investigate the effects beyond NPDR and into PDR. In this experiment, retinal vascular observation with fundoscopy revealed that the DM groups had atrophied retinal vessels compared to the INT group (Fig. 7a). However, there were limitations in quantitatively comparing these findings. Additionally, this study was unable to perform optical coherence tomography imaging of retinal changes or angiography to assess retinal vascular leakage. This limitation was due to the development of cataracts from DM progression, which hindered the ability to conduct these examinations.

In conclusion, mTOR inhibitor pretreatment of MSCs enhanced their therapeutic effect against early DR. Both everolimus- and sirolimus-pretreated MSCs preserved retinal function and structure more effectively than untreated MSCs. Although everolimus showed a nonsignificant trend toward better flicker ERG outcomes, both pretreatment strategies similarly protected the outer retinal layers histologically, without a significant difference between them.

## Supplementary Material

Supplement 1

## References

[1] Brownlee M . The pathobiology of diabetic complications: a unifying mechanism. *Diabetes*. 2005; 54(6): 1615–1625. 15919781 10.2337/diabetes.54.6.1615

[2] Sadikan MZ, Abdul Nasir NA, Lambuk L, et al. Diabetic retinopathy: a comprehensive update on in vivo, in vitro and ex vivo experimental models. *BMC Ophthalmol*. 2023; 23(1): 421. 37858128 10.1186/s12886-023-03155-1 PMC10588156

[3] Wang W, Lo ACY. Diabetic retinopathy: pathophysiology and treatments. *Int J Mol Sci*. 2018; 19(6): 1816. 29925789 10.3390/ijms19061816 PMC6032159

[4] Hammes H-P, Lin J, Renner O, et al. Pericytes and the pathogenesis of diabetic retinopathy. *Diabetes*. 2002; 51(10): 3107–3112. 12351455 10.2337/diabetes.51.10.3107

[5] Gomułka K, Ruta M. The role of inflammation and therapeutic concepts in diabetic retinopathy—a short review. *Int J Mol Sci*. 2023; 24(2): 1024. 36674535 10.3390/ijms24021024 PMC9864095

[6] Biswas S, Chakrabarti S. Pathogenetic mechanisms in diabetic retinopathy: from molecules to cells to tissues. In: Kartha CC, Ramachandran S, Pillai RM, eds. *Mechanisms of Vascular Defects in Diabetes Mellitus*. Cham: Springer; 2017: 209–247.

[7] Sadikan MZ, Abdul Nasir NA. Diabetic retinopathy: emerging concepts of current and potential therapy. *Naunyn Schmiedebergs Arch Pharmacol*. 2023; 396(12): 3395–3406. 37401966 10.1007/s00210-023-02599-y

[8] Cox JT, Eliott D, Sobrin L. Inflammatory complications of intravitreal anti-VEGF injections. *J Clin Med*. 2021; 10(5): 981. 33801185 10.3390/jcm10050981 PMC7957879

[9] Patel D, Patel SN, Chaudhary V, Garg SJ. Complications of intravitreal injections: 2022. *Curr Opin Ophthalmol*. 2022; 33(3): 137–146. 35266893 10.1097/ICU.0000000000000850

[10] Nguyen QD, Ibrahim MA, Watters A, et al. Ocular tolerability and efficacy of intravitreal and subconjunctival injections of sirolimus in patients with non-infectious uveitis: primary 6-month results of the SAVE Study. *J Ophthalmic Inflamm Infect*. 2013; 3(1): 32. 23514595 10.1186/1869-5760-3-32 PMC3610181

[11] Tsai C-H, Hoang LN, Lin CC, et al. Evaluation of topical and subconjunctival injection of hyaluronic acid-coated nanoparticles for drug delivery to posterior eye. *Pharmaceutics*. 2022; 14(6): 1253. 35745825 10.3390/pharmaceutics14061253 PMC9228085

[12] Jo HH, Goh YS, Kim HJ, et al. Tacrolimus improves therapeutic efficacy of umbilical cord blood-derived mesenchymal stem cells in diabetic retinopathy by suppressing DRP1-mediated mitochondrial fission. *Antioxidants*. 2023; 12(9): 1727. 37760030 10.3390/antiox12091727 PMC10525315

[13] Kim H, Goh Y-S, Park S-E, et al. Preventive effects of exosome-rich conditioned medium from amniotic membrane-derived mesenchymal stem cells for diabetic retinopathy in rats. *Transl Vis Sci Technol*. 2023; 12(8): 18. 10.1167/tvst.12.8.18 PMC10461646 37610767

[14] Kang N, Jung JS, Hwang J, et al. Beneficial effect of sirolimus-pretreated mesenchymal stem cell implantation on diabetic retinopathy in rats. *Biomedicines*. 2024; 12(2): 383. 38397985 10.3390/biomedicines12020383 PMC10886997

[15] Chen Y, Yao G, Tong J, et al. MSC-derived small extracellular vesicles alleviate diabetic retinopathy by delivering miR-22-3p to inhibit NLRP3 inflammasome activation. *Stem Cells*. 2024; 42(1): 64–75. 37847598 10.1093/stmcls/sxad078

[16] Yang Z, Li K, Yan X, Dong F, Zhao C. Amelioration of diabetic retinopathy by engrafted human adipose-derived mesenchymal stem cells in streptozotocin diabetic rats. *Graefes Arch Clin Exp Ophthalmol*. 2010; 248(10): 1415–1422. 20437245 10.1007/s00417-010-1384-z

[17] Zhang W, Wang Y, Kong J, Dong M, Duan H, Chen S. Therapeutic efficacy of neural stem cells originating from umbilical cord-derived mesenchymal stem cells in diabetic retinopathy. *Sci Rep*. 2017; 7(1): 408. 28341839 10.1038/s41598-017-00298-2 PMC5412648

[18] Zhao K, Liu J, Dong G, et al. Preliminary research on the effects and mechanisms of umbilical cord‑derived mesenchymal stem cells in streptozotocin‑induced diabetic retinopathy. *Int J Mol Med*. 2020; 46(2): 849–858. 32626946 10.3892/ijmm.2020.4623

[19] Kramerov AA, Ljubimov AV. Stem cell therapies in the treatment of diabetic retinopathy and keratopathy. *Exp Biol Med (Maywood)*. 2016; 241(6): 559–568. 26454200 10.1177/1535370215609692 PMC4950324

[20] Kern S, Eichler H, Stoeve J, Kluter H, Bieback K. Comparative analysis of mesenchymal stem cells from bone marrow, umbilical cord blood, or adipose tissue. *Stem Cells*. 2006; 24(5): 1294–1301. 16410387 10.1634/stemcells.2005-0342

[21] Sun F, Sun Y, Zhu J, et al. Mesenchymal stem cells-derived small extracellular vesicles alleviate diabetic retinopathy by delivering NEDD4. *Stem Cell Res Ther*. 2022; 13(1): 293. 35841055 10.1186/s13287-022-02983-0 PMC9284871

[22] Lechner J, Medina RJ, Lois N, Stitt AW. Advances in cell therapies using stem cells/progenitors as a novel approach for neurovascular repair of the diabetic retina. *Stem Cell Res Ther*. 2022; 13(1): 388. 35907890 10.1186/s13287-022-03073-x PMC9338609

[23] Ludwig PE, Freeman SC, Janot AC. Novel stem cell and gene therapy in diabetic retinopathy, age related macular degeneration, and retinitis pigmentosa. *Int J Retina Vitreous*. 2019; 5(1): 7. 30805203 10.1186/s40942-019-0158-y PMC6373096

[24] Park SS . Cell therapy applications for retinal vascular diseases: diabetic retinopathy and retinal vein occlusion. *Invest Ophthalmol Vis Sci*. 2016; 57(5): ORSFj1–ORSFj10. 27116667 10.1167/iovs.15-17594

[25] Almahasneh F, Abu-El-Rub E, Khasawneh RR, Almazari R. Effects of high glucose and severe hypoxia on the biological behavior of mesenchymal stem cells at various passages. *World J Stem Cells*. 2024; 16(4): 434. 38690519 10.4252/wjsc.v16.i4.434 PMC11056633

[26] Kroemer G, Mariño G, Levine B. Autophagy and the integrated stress response. *Mol Cell*. 2010; 40(2): 280–293. 20965422 10.1016/j.molcel.2010.09.023 PMC3127250

[27] Guan J-L, Simon AK, Prescott M, et al. Autophagy in stem cells. *Autophagy*. 2013; 9(6): 830–849. 23486312 10.4161/auto.24132 PMC3672294

[28] Usategui-Martin R, Puertas-Neyra K, Galindo-Cabello N, et al. Retinal neuroprotective effect of mesenchymal stem cells secretome through modulation of oxidative stress, autophagy, and programmed cell death. *Invest Ophthalmol Vis Sci*. 2022; 63(4): 27. 10.1167/iovs.63.4.27 PMC9055551 35486068

[29] Klawitter J, Nashan B, Christians U. Everolimus and sirolimus in transplantation-related but different. *Exp Opin Drug Safety*. 2015; 14(7): 1055–1070. 10.1517/14740338.2015.1040388 PMC6053318 25912929

[30] Chiu HY, Tsay YG, Hung SC. Involvement of mTOR-autophagy in the selection of primitive mesenchymal stem cells in chitosan film 3-dimensional culture. *Sci Rep*. 2017; 7(1): 10113. 28860574 10.1038/s41598-017-10708-0 PMC5578982

[31] Li Z-h, Wang Y-l, Wang H-j, Wu J-h, Tan Y-z. Rapamycin-preactivated autophagy enhances survival and differentiation of mesenchymal stem cells after transplantation into infarcted myocardium. *Stem Cell Rev Rep*. 2020; 16: 344–356. 31927699 10.1007/s12015-020-09952-1 PMC7152587

[32] Wang B, Lin Y, Hu Y, et al. mTOR inhibition improves the immunomodulatory properties of human bone marrow mesenchymal stem cells by inducing COX-2 and PGE(2). *Stem Cell Res Ther*. 2017; 8(1): 292. 29287601 10.1186/s13287-017-0744-6 PMC5747167

[33] Kim YC, Guan K-L. mTOR: a pharmacologic target for autophagy regulation. *J Clin Invest*. 2015; 125(1): 25–32. 25654547 10.1172/JCI73939 PMC4382265

[34] Eaton JS, Miller PE, Bentley E, Thomasy SM, Murphy CJ. The SPOTS system: an ocular scoring system optimized for use in modern preclinical drug development and toxicology. *J Ocul Pharmacol Ther*. 2017; 33(10): 718–734. 29239680 10.1089/jop.2017.0108

[35] De Simone U, Spinillo A, Caloni F, Gribaldo L, Coccini T. Neuron-like cells generated from human umbilical cord lining-derived mesenchymal stem cells as a new in vitro model for neuronal toxicity screening: using magnetite nanoparticles as an example. *Int J Mol Sci*. 2019; 21(1): 271. 31906090 10.3390/ijms21010271 PMC6982086

[36] Kacham S, Bhure TS, Eswaramoorthy SD, et al. Human umbilical cord-derived mesenchymal stem cells promote corneal epithelial repair in vitro. *Cells*. 2021; 10(5): 1254. 34069578 10.3390/cells10051254 PMC8160941

[37] Saha B, Roy A, Beltramo E, Sahoo OS. Stem cells and diabetic retinopathy: from models to treatment. *Mol Biol Rep*. 2023; 50(5): 4517–4526. 36842153 10.1007/s11033-023-08337-0

[38] Kuiper EJ, Rv Z, Roestenberg P, et al. Connective tissue growth factor is necessary for retinal capillary basal lamina thickening in diabetic mice. *J Histochem Cytochem*. 2008; 56(8): 785–792. 18474939 10.1369/jhc.2008.950980 PMC2443606

[39] Van Geest RJ, Lesnik-Oberstein SY, Tan HS, et al. A shift in the balance of vascular endothelial growth factor and connective tissue growth factor by bevacizumab causes the angiofibrotic switch in proliferative diabetic retinopathy. *Br J Ophthalmol*. 2012; 96(4): 587–590. 22289291 10.1136/bjophthalmol-2011-301005 PMC3308470

[40] Adak S, Magdalene D, Deshmukh S, Das D, Jaganathan BG. A review on mesenchymal stem cells for treatment of retinal diseases. *Stem Cell Rev Rep*. 2021; 17(4): 1154–1173. 33410097 10.1007/s12015-020-10090-x PMC7787584

[41] Liang J, Dai W, Xue S, Wu F, Cui E, Pan R. Recent progress in mesenchymal stem cell-based therapy for acute lung injury. *Cell Tissue Banking*. 2024; 25(2): 677–684. 38466563 10.1007/s10561-024-10129-0

[42] Mabotuwana NS, Rech L, Lim J, et al. Paracrine factors released by stem cells of mesenchymal origin and their effects in cardiovascular disease: a systematic review of pre-clinical studies. *Stem Cell Rev Rep*. 2022; 18(8): 2606–2628. 35896860 10.1007/s12015-022-10429-6 PMC9622561

[43] Fiori A, Terlizzi V, Kremer H, et al. Mesenchymal stromal/stem cells as potential therapy in diabetic retinopathy. *Immunobiology*. 2018; 223(12): 729–743. 29402461 10.1016/j.imbio.2018.01.001

[44] Rani S, Ryan AE, Griffin MD, Ritter T. Mesenchymal stem cell-derived extracellular vesicles: toward cell-free therapeutic applications. *Mol Ther*. 2015; 23(5): 812–823. 25868399 10.1038/mt.2015.44 PMC4427881

[45] Strettoi E, Novelli E, Mazzoni F, Barone I, Damiani D. Complexity of retinal cone bipolar cells. *Prog Retin Eye Res*. 2010; 29(4): 272–283. 20362067 10.1016/j.preteyeres.2010.03.005 PMC2878852

[46] McAnany JJ, Park JC, Chau FY, Leiderman YI, Lim JI, Blair NP. Amplitude loss of the high-frequency flicker electroretinogram in early diabetic retinopathy. *Retina*. 2019; 39(10): 2032–2039. 30024576 10.1097/IAE.0000000000002262 PMC6335196

[47] Arias-Alvarez M, Tomas-Grasa C, Sopena-Pinilla M, et al. Electrophysiological findings in long-term type 1 diabetes patients without diabetic retinopathy using different ERG recording systems. *Sci Rep*. 2024; 14(1): 3520. 38347052 10.1038/s41598-024-54099-5 PMC10861544

[48] McAnany JJ, Persidina OS, Park JC. Clinical electroretinography in diabetic retinopathy: a review. *Surv Ophthalmol*. 2022; 67(3): 712–722. 34487740 10.1016/j.survophthal.2021.08.011 PMC9158180

[49] Cone RA . The rat electroretinogram. I. Contrasting effects of adaptation on the amplitude and latency of the b-wave. *J Gen Physiol*. 1964; 47(6): 1089–1105. 14192547 10.1085/jgp.47.6.1089 PMC2195382

[50] Schaeppi U, Krinke G, Fink X, Hofer R, Duennenberger D. Electroretinography in rats. *Agents Actions*. 1988; 24(3–4): 395–402. 3177098 10.1007/BF02028299

[51] Tavares Ferreira J, Proenca R, Alves M, et al. Retina and choroid of diabetic patients without observed retinal vascular changes: a longitudinal study. *Am J Ophthalmol*. 2017; 176: 15–25. 28057456 10.1016/j.ajo.2016.12.023

[52] Tonade D, Kern TS. Photoreceptor cells and RPE contribute to the development of diabetic retinopathy. *Prog Retin Eye Res*. 2021; 83: 100919. 33188897 10.1016/j.preteyeres.2020.100919 PMC8113320

[53] Kern TS, Berkowitz BA. Photoreceptors in diabetic retinopathy. *J Diabetes Invest*. 2015; 6(4): 371–380. 10.1111/jdi.12312 PMC4511295 26221514

[54] Park S-H, Park J-W, Park S-J, et al. Apoptotic death of photoreceptors in the streptozotocin-induced diabetic rat retina. *Diabetologia*. 2003; 46: 1260–1268. 12898017 10.1007/s00125-003-1177-6

[55] Énzsöly A, Szabó A, Kántor O, et al. Pathologic alterations of the outer retina in streptozotocin-induced diabetes. *Invest Ophthalmol Vis Sci*. 2014; 55(6): 3686–3699. 24845643 10.1167/iovs.13-13562

[56] De Becker A, Riet IV. Homing and migration of mesenchymal stromal cells: how to improve the efficacy of cell therapy? *World J Stem Cells*. 2016; 8(3): 73–87. 27022438 10.4252/wjsc.v8.i3.73 PMC4807311

[57] Luo C, Zhang YS, Zhang MX, et al. Everolimus versus sirolimus for angiomyolipoma associated with tuberous sclerosis complex: a multi-institutional retrospective study in China. *Orphanet J Rare Dis*. 2021; 16(1): 299. 34217357 10.1186/s13023-021-01932-z PMC8254951

[58] MacKeigan JP, Krueger DA. Differentiating the mTOR inhibitors everolimus and sirolimus in the treatment of tuberous sclerosis complex. *Neuro Oncol*. 2015; 17(12): 1550–1559. 26289591 10.1093/neuonc/nov152 PMC4633932

[59] Gong C-Y, Lu B, Hu Q-W, Ji L-L. Streptozotocin induced diabetic retinopathy in rat and the expression of vascular endothelial growth factor and its receptor. *Int J Ophthalmol*. 2013; 6(5): 573. 24195027 10.3980/j.issn.2222-3959.2013.05.03 PMC3808899

[60] Polewik K, Kosek M, Jamrozik D, et al. Rodent models of diabetic retinopathy as a useful research tool to study neurovascular cross-talk. *Biology (Basel)*. 2023; 12(2): 262. 36829539 10.3390/biology12020262 PMC9952991

