# Supplementary material for: Efficacy of Subconjunctivally Applied Everolimus- and Sirolimus-Pretreated MSCs in Preventing Diabetic Retinopathy
Source: Transl Vis Sci Technol. 2025 Sep 15;14(9):19. doi: 10.1167/tvst.14.9.19 (PMC12442941; doi:10.1167/tvst.14.9.19)
Supplement: Supplement 1 [file tvst-14-9-19_s001.docx]

**Supplementary Materials**

**Table S1.** Sequences of primers used for qPCR

| Gene | Identification | Sequence (5’-3’) | Size (bp) | Accession  number |
| --- | --- | --- | --- | --- |
| *NT5E* | Sense | CTCTTGCAACACCCATGTGC | 225 | NM_001204813.2 |
|  | Antisense | ACAGCTAATGCCGTGTGTCA |  |  |
| *THY1* | Sense | ATCCAAAAGCATCGGCAGGA | 243 | NM_006288.5 |
|  | Antisense | CCTTCCTCCCCCAAATTCCC |  |  |
| *ENG* | Sense | TACCCATACCCAAAACCGGC | 222 | NM_000118.4 |
|  | Antisense | TGTACCAGAGTGCAGCAGTG |  |  |
| *ACTB* | Sense | CCACCATGTACCCTGGCATT | 189 | NM_001101.5 |
|  | Antisense | CGGACTCGTCATACTCCTGC |  |  |

**Table S2.** BW values at each measurement point: week 0, week 2, and week 8 post STZ injection.

|  | INT | DM | MSC | MSC-E | MSC-S |
| --- | --- | --- | --- | --- | --- |
| 0w | 332.5±15.3 | 326.4±12.9 | 333.1±8.1 | 332.0±16.6 | 325.6±15.9 |
| 2w | 422.3±16.5 | 309.9±27.6 | 309.1±15.3 | 288.3±20.2 | 303.8±32.1 |
| 8w | 527.8±20.7 | 295.3±41.5 | 279.4±15.9 | 267.5±25.1 | 288.8±41.2 |

INT: intact control group. DM: diabetes mellitus-induced group. MSC: diabetes mellitus-induced group with subconjunctival MSC injection. MSC-E: diabetes mellitus-induced group with subconjunctival everolimus-pretreated MSC injection. MSC-S: diabetes mellitus-induced group with subconjunctival sirolimus-pretreated MSC injection. Values are expressed as mean ± SD, with units in grams.

**Table S3.** BG values at each measurement point: week 0, week 2, and week 8 post STZ injection.

|  | INT | DM | MSC | MSC-E | MSC-S |
| --- | --- | --- | --- | --- | --- |
| 0w | 115.8±12.4 | 113.1±19.2 | 117.4±11.3 | 114.5±13.0 | 117.0±9.78 |
| 2w | 126.8±8.2 | 379.9±39.6 | 429.5±37.4 | 446.5±29.2 | 418.0±16.7 |
| 8w | 102.8±11.6 | 387.8±106.7 | 433.9±53.5 | 464.8±28.9 | 423.4±66.7 |

INT: intact control group. DM: diabetes mellitus-induced group. MSC: diabetes mellitus-induced group with subconjunctival MSC injection. MSC-E: diabetes mellitus-induced group with subconjunctival everolimus-pretreated MSC injection. MSC-S: diabetes mellitus-induced group with subconjunctival sirolimus-pretreated MSC injection. Values are expressed as mean ± SD, with units in milligrams per deciliter (mg/dL).
